# Vitamin D Status Over Time and Cognitive Function in Norwegian Older Adults: A Prospective Cohort of the HUNT Study

**DOI:** 10.1007/s12603-022-1867-8

**Published:** 2022-12-07

**Authors:** Ernest Obeng Asante, X.-M. Mai, R.S. Eldholm, H.K. Skjellegrind, M. Kolberg, B.M. Brumpton, G. Selbœk, Y. Chen, Y.-Q. Sun

**Affiliations:** 1 Department of Clinical and Molecular Medicine, NTNU, Norwegian University of Science and Technology, Trondheim, Norway; 2 Center for Oral Health Services and Research Mid-Norway (TkMidt), Trondheim, Norway; 3 Department of Public Health and Nursing, NTNU, Norwegian University of Science and Technology, Trondheim, Norway; 4 Department of Neuromedicine and Movement Science, NTNU, Norwegian University of Science and Technology, Trondheim, Norway; 5 Department of Geriatrics, Clinic of Medicine, St. Olavs Hospital, Trondheim, Norway; 6 HUNT Research Centre, Department of Public Health and Nursing, NTNU, Norwegian University of Science and Technology, Levanger, Norway; 7 Levanger Hospital, Nord-Trøndelag Hospital Trust, Levanger, Norway; 8 K.G. Jebsen Center for Genetic Epidemiology, Department of Public Health and Nursing, NTNU, Norwegian University of Science and Technology, Trondheim, Norway; 9 Clinic of Medicine, St. Olavs hospital Trondheim University Hospital, Trondheim, Norway; 10 Norwegian National Centre for Ageing and Health, Vestfold Hospital Trust, Tønsberg, Norway; 11 Department of Geriatric Medicine, Oslo University Hospital, Oslo, Norway; 12 Institute of Clinical Medicine, Faculty of Medicine, University of Oslo, Oslo, Norway; 13 School of Epidemiology and Public Health, Faculty of Medicine, University of Ottawa, Ottawa, Ontario, Canada; 14 Department of Pathology, Clinic of Laboratory Medicine, St. Olavs Hospital, Trondheim, Norway

**Keywords:** 25(OH)D, neurocognitive disorders, dementia, HUNT study, MCI

## Abstract

**Background:**

There is conflicting evidence regarding the association between vitamin D status and cognitive function in population studies. The use of one-time vitamin D measurement in cognitive health studies may not reflect long-term vitamin D status in the body.

**Objective:**

We aimed to examine the relationship of vitamin D status measured over time with the risk of neurocognitive disorders (NCDs) in Norwegian older adults.

**Design:**

Prospective cohort study.

**Setting:**

Regional, Trøndelag Health Study.

**Participants:**

This study followed a random cohort of 717 participants from HUNT2 (1995–97) and HUNT3 (2006–08) to HUNT4 70+ (2017–19). The mean age at HUNT4 70+ was 77.7 years.

**Methods:**

Seasonal-standardized serum 25-hydroxyvitamin D [25(OH)D] levels in HUNT2 and HUNT3 were averaged and used as either a categorical variable (<50 and ≥50 nmol/L) or a continuous variable (per 25 nmol/L decrease). In the cohort aged 70 years or over (HUNT4 70+), NCDs consisting of mild cognitive impairment (MCI) and dementia were diagnosed by clinical experts according to the DSM-5 criteria. Logistic and linear regression models were used to estimate odds ratios (ORs) and regression coefficients (beta) with 95% confidence intervals (CIs) to assess the relationship between 25(OH) D levels and the risk of NCDs or the Montreal Cognitive Assessment (MoCA) score.

**Results:**

In total, 347 (48.4%) had NCDs in HUNT4, with 33.3% having MCI and 15.1% having dementia. Compared with participants with serum 25(OH)D ≥50 nmol/L, those with 25(OH)D <50 nmol/L had a similar risk of NCDs (OR 1.05, 95% CI 0.76 to 1.46). No association was observed with the risk of MCI (OR 1.01, 95% CI 0.71 to 1.44) or dementia (OR 1.16, 95% CI 0.70 to 1.92), respectively. In a subsample of participants evaluated with the MoCA (n=662), a 25 nmol/L decrease in serum 25(OH)D was not associated with a change in MoCA score (beta 0.33, 95% CI −0.17 to 0.85).

**Conclusion:**

Vitamin D insufficiency defined by two times measurements of serum 25(OH)D with a 10-year interval was not associated with the risk of NCDs in a cohort of older Norwegian adults. Future studies utilizing multiple vitamin D measurements with a longer follow-up duration and larger sample size are warranted.

## Introduction

**N**eurocognitive disorders (NCDs) in older adults have become a public health priority amidst an increasingly aging population. There were an estimated 57.4 million dementia cases globally in 2019, with a projected increase to 152.8 million cases in 2050 (1). Mild cognitive impairment (MCI) is characterized by a higher-than-expected cognitive decline for an individual, taking age or education level into account, with minimal impact on daily activities (2). More than half of MCI cases progress to dementia within five years (2). A recent Norwegian study has shown a high prevalence of MCI and dementia. Today, there are more than 100,000 dementia cases in Norway and cases are predicted to more than double by 2050 (3). Dementia is a chronic condition that poses a significant burden on patients and families with overwhelming responsibilities on caregivers. The cost of care also increases with disease severity (4). Since there is no cure, preventing or delaying disease onset is essential to the socio-economic impact. Identified modifiable risk factors, including low education, hearing loss, smoking, depression, physical inactivity, hypertension, obesity, and diabetes, account for about 40% of dementia cases worldwide (5). Identification of new modifiable risk factors could improve the prevention further.

Vitamin D receptor, a nuclear steroid receptor required for vitamin D to exhibit its effect, is widely distributed in the brain (6). Both animal and in vitro experimental studies have shown neuroprotective and anti-inflammatory effects of serum 25-hydroxyvitamin D [25(OH)D] on the brain and cognition (7, 8). Such effects have been demonstrated in mice with the ability of 25(OH)D to clear amyloid plaque deposits in the brain, a hallmark of Alzheimer's disease (8). Regardless, the role of 25(OH)D in cognitive function is inconclusive in population studies. In some prospective cohort studies, associations have been reported between low 25(OH)D (i.e., <50 nmol/L) and cognitive decline or the risk of dementia (9, 10, 11, 12), supported by pooled estimates from two meta-analyses (13, 14). However, other prospective studies reported no such associations (15, 16, 17). Similarly, causality is yet to be established through intervention studies and Mendelian randomization studies (18, 19).

The absence of repeated 25(OH)D measures in cognitive health studies has been considered a drawback but has not been explored (13, 14). One-time measurement may not reflect long-term 25(OH)D status in the body. Hence, this study aimed to examine the relationship of vitamin D status measured over time with the risk of NCDs in older Norwegian adults.

## Method

### Study Design and Populations

The study population was drawn from the Tr0ndelag Health Study (HUNT Study) — a large and comprehensive health study in Norway. These surveys have been conducted in series from HUNT1 (1984–1986) to HUNT4 (2017–19). Residents in the Nord-Trøndelag region in Norway aged ≥20 years were invited to participate in these surveys. Participants answered a range of health and lifestyle-related questions and attended a clinical examination for each survey (20).

**Figure 1:**
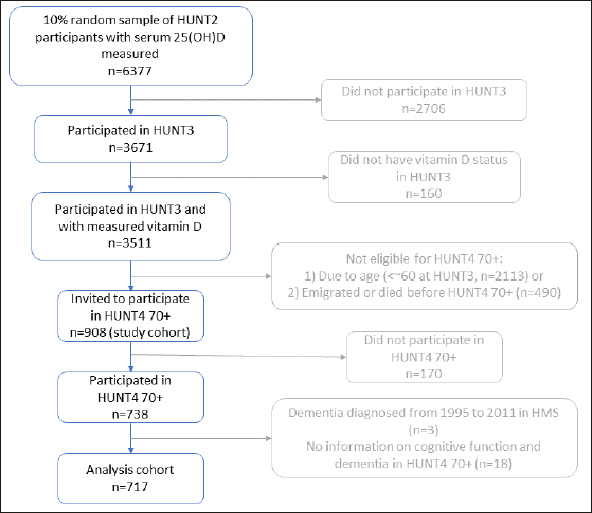
Flow chart of selection criteria for the analysis cohort in the HUNT Study

We selected a 10% random sample (n=6377) from HUNT2 participants (1995–97) and measured their serum 25(OH) D level, and the methodology has been described in detail previously (21). Among them, 3673 (57.6%) participated in HUNT3 (2006–08), and 3511 had serum 25(OH)D measured in HUNT3. Of the 3511 participants, 908 (the study cohort) were invited to HUNT4 70+, and 738 participated. HUNT4 70+ invited HUNT4 participants aged 70 years or older (3). We excluded dementia cases diagnosed from 1995 to 2011 in the Health and Memory Study (n=3) (22) and those with no information on cognitive function in HUNT4 70+ (n=18). The current analysis was based on data from 717 individuals (the analysis cohort) who had complete information on serum 25(OH)D levels in HUNT2 and HUNT3 with cognitive function evaluated in HUNT4 70+ and dementia-free from 1995 to 2011. The mean age of the 717 participants in HUNT4 70+ was 77.7 years. A flowchart outlining this selection process is presented in Figure 1.

Analysis cohort comprised of 717 individuals who had complete information on serum 25(OH)D levels in both HUNT2 and HUNT3 and cognitive function evaluated in HUNT4 70+, as well as dementia-free at baseline. HMS, Health and Memory Study; HUNT, Trøndelag Health Study.

### Serum 25(OH)D Measurements

Blood samples collected in HUNT2 and HUNT3 were stored at −80°C until analysis. Serum 25(OH)D levels in both HUNT surveys were measured using LIAISON 25-OH vitamin D TOTAL (DiaSorin, Saluggia, Italy), a fully automated antibody-based chemiluminescence assay with a detection range of 10–375 nmol/L. Serum 25(OH)D represents the vitamin D status in the body and reflects vitamin D obtained from sunlight exposure, food intake and supplementation (23).

The geographical position of Norway implies seasonal fluctuations in 25(OH)D levels. A cosinor model based on the month of blood draw was used to calculate the seasonal-standardized 25(OH)D level (nmol/L) (24, 25) — which represents the annual average value of 25(OH)D for each participant. The seasonal-standardized 25(OH)D levels of HUNT2 and HUNT3 were averaged to denote 25(OH)D levels over time. We used the average seasonal-standardized serum 25(OH)D level as categorical and continuous variables. Serum 25(OH)D categorization followed cut-off values of <50 nmol/L as insufficiency and ≥50 nmol/L as sufficiency and per 25 nmol/L decrease as a continuous variable (26, 27). To account for changes in 25(OH)D levels over time, we also categorized seasonal-standardized 25(OH)D as long-term insufficiency (<50 nmol/L in both HUNT2 and HUNT3), long-term sufficiency (≥50 nmol/L in both HUNT2 and HUNT3), and varying levels (change from <50 nmol/L to ≥50 nmol/L and vice versa between HUNT2 and HUNT3) for additional analysis. We performed further analyses using four groups of average seasonal-standardized serum 25(OH)D (nmol/L, <30, 30–49.9, 50–74.9, ≥75) (26).

### Covariates

HUNT3 was regarded as the baseline in this study. Other baseline variables in HUNT3 were collected through questionnaires and clinical examination. Sociodemographic variables included age (as a continuous variable), sex, occupation (defined by Erikson Goldthorpe Portocarero social class scheme ranging from high to low social status: EGP class I to VII) (28), and marital status (unmarried, married, and others). Body mass index (BMI), calculated as weight divided by squared height, was classified into underweight or normal (<25.0 kg/m^2^) (only one participant with BMI<18.5 kg/m^2^), overweight (25.0–29.9 kg/m^2^), and obesity (≥30.0 kg/m^2^). Lifestyle factors included smoking status in packyears [never smokers, former smokers (0–10, 10.1–20, and >20 pyrs), current smokers (0–10, 10.1–20 and >20 pyrs)], alcohol consumption per month (never, 1–4 times, or ≥5 times) and physical activity levels (inactive, low, moderate, or high) (29, 30). Other health-related baseline variables include diabetes (yes, no) and hypertension (yes, no). Participants were defined as having diabetes if they answered yes to a question: ‘Have you had or do you have diabetes?' and/or had a non-fasting blood glucose level above 11 mmol/L. Participants were defined as having hypertension if they took medication for hypertension and/or had systolic blood pressure ≥140 mm Hg and/or diastolic blood pressure ≥90 mm Hg. Serum cholesterol level was classified into desirable (<5.2 mmol/L), borderline (5.2 – 6.2 mmol/L), and high (>6.2 mmol/L). Information on depression was collected using the Hospital Anxiety and Depression Scale (HADS) and categorized as non-cases (HADS≤7) and cases (HADS≥8) (31). Kidney function was represented by serum creatinine levels (µmol/L) as a continuous variable. Participants with missing information on covariates were regarded as an “unknown” category for each variable and included in the statistical analyses.

### Neurocognitive Disorders in HUNT4 70+

Participants in HUNT4 70+ were grouped into no cognitive impairment, MCI (amnestic or non-amnestic MCI), and dementia after a thorough clinical evaluation by clinical experts. Participants diagnosed with MCI or dementia were combined as NCDs. The diagnosis was based on testing in various cognitive domains and structured caregiver questionnaires according to the Diagnostic and Statistical Manual of Mental Disorders, Fifth Edition (DSM-5) criteria (3). Thus, NCDs assessment covered cognition, function in daily life, neuropsychiatric symptoms, subjective cognitive decline, symptom debut, and course of the condition. Subsequent diagnoses were made by applying standard diagnostic criteria for MCI (mild neurocognitive disorder), dementia (major neurocognitive disorder) and all the dementia subtypes according to the DSM-5 (32). A detailed description of assessments and procedures for diagnosing NCDs in HUNT4 70+ utilizing the DSM-5 framework can be found in the study by Gjøra et al. (3).

The Montreal Cognitive Assessment (MoCA) scale was used as one of the assessment tools for cognition in HUNT4 70+. The related score served as a secondary outcome for this study. The MoCA is a well-established, multidomain cognitive screening instrument for evaluating age-related cognitive decline (33). The MoCA scale tests memory, visuospatial and executive functions, naming, attention, abstraction, language and orientation. The score ranges from 0 to 30, and higher scores suggest better cognitive function. Some participants were not assessed with MoCA due to incomplete tests or severe cognitive impairment (n=55) (3).

### Statistical Analyses

The distribution of baseline covariates in HUNT3 was presented according to the average seasonal-standardized serum 25(OH)D categories (<50 and ≥50 nmol/L). Serum 25(OH) D was used as a categorical variable (<50 and ≥50 nmol/L) or a continuous variable (25 nmol/L decrease in 25(OH)D). The relationship between serum 25(OH)D and the risk of NCDs was evaluated using logistic regression models to estimate odds ratios (ORs) with 95% confidence intervals (CIs). We further calculated ORs for MCI and dementia in relation to serum 25(OH)D using multinomial logistic regression. The relationship between serum 25(OH)D levels and MoCA score was accessed by linear regression to estimate the regression coefficient (beta). In a sensitivity analysis, we evaluated the association of changes in 25(OH)D levels over 10 years with the risk of NCDs.

In multivariable regression analysis, we adjusted for age (as a continuous variable), sex, BMI, occupation, marital status, smoking status in packyears, alcohol consumption and physical activity in the main model (Model 1). Additional analyses were performed to adjust for diabetes, blood pressure, serum cholesterol, depression, and serum creatinine (as a continuous variable) in Model 2. These potential confounders were included based on previous studies on the association between 25(OH)D and NCDs (5, 14, 15). Continuous variables such as serum 25(OH)D level, age, and serum creatinine level were used without additional transformations for analysis despite deviations from a normal distribution. Deviations were mostly minor except for age.

We addressed potential bias due to missing data in covariates (the “Unknown” category in Table 1) using multiple imputation by chained equations, assuming data in covariates were missing at random. Based on recommendations (34), analysis was executed on 10 imputed data sets and results represented the averaged estimates.

**Table 1:** Baseline characteristics in the HUNT3 survey of participants overall and stratified by average seasonal-standardized serum 25(OH)D levels of HUNT2 and HUNT3

	**Average seasonal-standardized serum 25(OH)D level (nmol/L)**		
	**<50 (n=324)**	**≥50 (n=393)**	**Total (n=717)**
Age (years)	66.5 ± 6.2	67.6 ± 6.1	67.1 ± 6.2
Sex			
Male	141 (43.5)	181 (46.1)	322 (44.9)
Female	183 (56.5)	212 (53.9)	395 (55.1)
Body mass index (kg/m^2^)			
Underweight or Normal (<25.0)	59 (18.2)	110 (28.0)	169 (23.6)
Overweight (25.0–29.9)	151 (46.6)	201(51.2)	352 (49.1)
Obesity (≥30.0)	113 (34.9)	81(20.6)	194 (27.1)
Unknown	1 (0.3)	1(0.3)	2 (0.3)
Occupation^†^			
EGP Class I	44 (13.6)	59 (15.0)	103 (14.4)
EGP Class II	43 (13.3)	78 (19.9)	121(16.9)
EGP Class III	95 (29.3)	100(25.5)	195 (27.2)
EGP Class IV	43 (13.3)	55 (14.0)	98 (13.7)
EGP Class V + VI	48 (14.8)	58 (14.8)	106 (14.8)
EGP Class VII	35 (10.8)	24 (6.1)	59 (8.2)
Unknown	16 (4.9)	19 (4.8)	35 (4.9)
Marital status			
Unmarried	16 (4.9)	12 (3.1)	28 (3.9)
Married	221 (68.2)	297 (75.6)	518 (72.3)
Others*	87 (26.9)	84 (21.4)	171 (23.9)
Smoking status in packyears (pyrs)			
Never smokers	135 (41.6)	176 (44.8)	311 (43.4)
Former smokers 0–10 pyrs	43 (13.3)	59 (15.0)	102 (14.2)
Former 10.1–20 pyrs	18 (5.6)	40 (10.2)	58 (8.1)
Former >20 pyrs	26 (8.0)	33 (8.4)	59 (8.2)
Current smokers 0–10 pyrs	5 (1.5)	9 (2.3)	14 (1.9)
Current 10.1–20 pyrs	13 (4.0)	13 (3.3)	26 (3.6)
Current >20 pyrs	25 (7.7)	21 (5.3)	46 (6.4)
Unknown	59 (18.2)	42 (10.7)	101 (14.1)
Alcohol consumption			
Never	21 (6.5)	23 (5.9)	44 (6.1)
1–4 times per month	248 (76.5)	278 (70.7)	526 (73.4)
≥ 5 times per month	49 (15.1)	81 (20.6)	130 (18.1)
Unknown	6 (1.9)	11 (2.8)	17 (2.4)
Physical activity			
Inactive	25 (7.7)	27 (6.9)	52 (7.3)
Low	41 (12.7)	44 (11.2)	85 (11.9)
Moderate	70 (21.6)	96 (24.4)	166 (23.2)
High	27 (8.3)	49 (12.5)	76 (10.6)
Unknown	161 (49.7)	177 (45.0)	338 (47.1)
Diabetes			
No	292 (90.1)	361 (91.9)	653 (91.1)
Yes	25 (7.7)	24 (6.1)	49 (6.8)
Unknown	7 (2.2)	8 (2.0)	15 (2.1)
Hypertension			
No	119 (36.7)	165 (42.0)	284 (39.6)
Yes	204 (64.0)	227 (57.8)	431 (60.1)
Unknown	1 (0.3)	1 (0.3)	2 (0.3)
Serum cholesterol (mmol/L)			
Desirable (<5.2)	86 (26.5)	123 (31.3)	209 (29.1)
Borderline (5.2 – 6.2)	113 (34.9)	147 (37.4)	260 (36.3)
High (>6.2)	117 (36.1)	115 (29.3)	232 (32.4)
Unknown	8 (2.5)	8 (2.0)	16 (2.2)
Depression (HADS)			
Non-cases (≤7)	244 (75.3)	323 (82.2)	567 (79.1)
Cases (≥8)	35 (10.8)	31(7.9)	66 (9.2)
Unknown	45 (13.9)	39 (9.9)	84 (11.7)
Serum creatinine [µmol/L]	68.7 ± 15.6	68.9 ± 16.1	68.8 ± 15.9
Data are given as the number of participants (column percentage) or mean ± 1SD.			

25(OH)D, 25-hydroxyvitamin D; EGP, Erikson Goldthorpe Portocarero social class scheme; HUNT, Trøndelag Health Study; HADS, The Hospital Anxiety and Depression Scale.

†Occupation: EGP Class I (administrative managers, politicians or academic professions), EGP Class II (occupations with shorter college and university degrees), EGP Class III (office and customer service occupations, sales, service and care professions), EGP Class IV (occupations in agriculture, forestry and fishing), EGP Class V + VI (craftsmen, process and machine operators or transport), EGP Class VII (occupations without education requirements). *Others (widow/widower, divorced, separated and unknown).

All Statistical analysis was performed with STATA/MP Version 17 (StataCorp LP, College Station, Texas).

## Results

The mean of seasonal-standardized serum 25(OH)D for the 717 participants was 49.1 nmol/L (SD 15.4) in HUNT2 and 57.7 nmol/L (SD 19.0) in HUNT3 (Supplementary figure 1). More participants had average seasonal-standardized serum 25(OH)D level of ≥50 nmol/L than <50 nmol/L (54.8% vs. 45.2%). Table 1 shows the HUNT3 baseline characteristics of the participants included in the current analysis. The mean age was 67.1 years and was slightly younger in participants with serum 25(OH)D level of <50 nmol/L compared to those with ≥50 nmol/L (66.5 vs. 67.6 years). Overall, participants with serum 25(OH)D levels <50 nmol/L had lower social class and a smaller portion were married, were more likely to have obesity, hypertension, and hypercholesterolemia compared with those with 25(OH)D ≥50 nmol/L.

Of the 717 participants, 347 (48.4%) had NCDs in HUNT4 70+, with 33.3% having MCI and 15.1% having dementia. Compared to participants with 25(OH)D level of ≥50 nmol/L, participants with 25(OH)D levels <50 nmol/L had a similar risk of NCDs (OR 1.05, 95% CI 0.76 – 1.46) after adjusting for potential confounders in the main model (Table 2, Model 1). Additional adjustments in Model 2 did not affect association estimates substantially. We observed no association between 25(OH)D levels <50 nmol/L and the risk of MCI or dementia in the main model (MCI: OR 1.01, 95% CI 0.71 – 1.44; dementia: OR 1.16, 95% CI 0.70 – 1.92). In addition, a 25 nmol/L decrease in serum 25(OH)D was not associated with the risk of NCDs (OR 0.97, 95% CI 0.74 – 1.27), nor with the risks of MCI (OR 1.06, 95% CI 0.79 – 1.44) and dementia (OR 0.78, 95% CI 0.52 – 1.17).

**Table 2:** Relationship between average seasonal-standardized serum 25(OH)D level of HUNT2 and HUNT3 and risk of NCDs in HUNT4 70+ (n=717)

**Average seasonal-standardized serum 25(OH) D (nmol/L)**	**Participants (n)**	**Cases (n)**	**Risk (%)**	**Crude OR (95% CI)**	**Adjusted OR (95% CI) Model 1**^†^	**Adjusted OR (95% CI) Model 2**^¥^
NCDs*						
Categorical						
<50	324	162	50.0	1.12 (0.84 – 1.51)	1.05 (0.76 – 1.46)	1.01 (0.72 – 1.41)
≥50	393	185	47.1	1.00 (Reference)	1.00 (Reference)	1.00 (Reference)
Continuous‡	717	347	48.4	1.09 (0.86 – 1.38)	0.97 (0.74 – 1.27)	0.94 (0.71 – 1.24)
Mild cognitive impairment (MCI)						
Categorical						
<50	324	109	33.6	1.08 (0.78 – 1.49)	1.01 (0.71 – 1.44)	0.98 (0.68 – 1.40)
≥50	393	130	33.1	1.00 (Reference)	1.00 (Reference)	1.00 (Reference)
Continuous†	717	239	33.3	1.17 (0.89 – 1.53)	1.06 (0.79 – 1.44)	1.03 (0.75 – 1.42)
Dementia						
Categorical						
<50	324	53	16.4	1.24 (0.81 – 1.90)	1.16 (0.70 – 1.92)	1.09 (0.64 – 1.83)
≥50	393	55	14.0	1.00 (Reference)	1.00 (Reference)	1.00 (Reference)
Continuous‡	717	108	15.1	0.94 (0.67 – 1.32)	0.78 (0.52 – 1.17)	0.73 (0.48 – 1.11)

25(OH)D, 25-hydroxyvitamin D; 95% CI, 95% confidence interval; NCDs, neurocognitive disorders; OR, odds ratio. *NCDs consisted of mild cognitive impairment (MCI) and dementia. †Model 1 was adjusted for age, sex, body mass index, occupation, marital status, smoking status in packyears, alcohol consumption and physical activity. ¥Model 2 was adjusted for diabetes, hypertension, serum cholesterol, depression, and kidney function (serum creatinine) in addition to Model 1. ‡per 25 nmol/L decrease in serum 25(OH)D.

Change in serum 25(OH)D levels over time (classified as long-term insufficiency, long-term sufficiency, and varying levels) was not associated with the risk of NCDs (Table 3). The adjusted ORs in the main model for the risk of NCDs in participants with long-term insufficiency and participants with varying 25(OH)D levels were 0.95 (95% 0.64 – 1.42) and 0.90 (95% CI 0.62 – 1.33), respectively. No association was observed between change in serum 25(OH)D levels over time and the risk of MCI or dementia, respectively.

**Table 3:** Relationship between changes in serum 25(OH)D levels over time and risk of NCDs in HUNT4 70+ (n=717)

**Seasonal-standardized serum 25(OH)D (nmol/L)**	**Participants (n)**	**Cases (n)**	**Risk (%)**	**Crude OR (95% CI)**	**Adjusted OR (95% CI) Model 1**^†^	**Adjusted OR (95% CI) Model 2**^¥^
NCDs*						
Long-term insufficiency <50	216	110	50.9	1.10 (0.77 – 1.58)	0.95 (0.64 – 1.42)	0.87 (0.57 – 1.31)
Long-term sufficiency ≥50	262	127	48.5	1.00 (Reference)	1.00 (Reference)	1.00 (reference)
Varying levels	239	110	46.0	0.91 (0.64 – 1.29)	0.90 (0.62 – 1.33)	0.88 (0.59 – 1.30)
Mild cognitive impairment (MCI)						
Long-term insufficiency <50	216	75	34.7	1.07 (0.72 – 1.60)	0.95 (0.62 – 1.47)	0.88 (0.56 – 1.38)
Long-term sufficiency ≥50	262	89	34.0	1.00 (Reference)	1.00 (Reference)	1.00 (Reference)
Varying levels	239	75	31.4	0.88 (0.60 – 1.30)	0.84 (0.55 – 1.27)	0.82 (0.53 – 1.26)
Dementia						
Long-term insufficiency <50	216	35	16.0	1.17 (0.69 – 1.98)	0.94 (0.51 - 1.73)	0.81 (0.43 – 1.55)
Long-term sufficiency ≥50	262	38	14.5	1.00 (Reference)	1.00 (Reference)	1.00 (Reference)
Varying levels	239	35	14.6	0.96 (0.57 – 1.62)	1.02 (0.56 – 1.86)	0.96 (0.51 – 1.80)

25(OH)D, 25-hydroxyvitamin D; 95% CI, 95% confidence interval; NCDs, neurocognitive disorders; OR odds ratio; *NCDs consisted of mild cognitive impairment (MCI) and dementia; †Model 1 was adjusted for age, sex, body mass index, occupation, marital status, smoking status in packyears, alcohol consumption and physical activity; ¥ Model 2 was adjusted for diabetes, blood pressure, cholesterol, depression, and kidney function(creatinine) in addition to Model 1.

In a subsample of participants assessed with MoCA (n= 662, Table 4 and Supplementary figure 2), 25(OH)D <50 nmol/L was not associated with the MoCA score (beta ∼0.06, 95% CI −0.67 – 0.56). Long-term insufficiency <50 nmol/L (beta 0.04, 95% CI −0.72 – 0.79) and the varying 25(OH)D levels (beta −0.09, 95% CI −0.81 – 0.63) were not related to MoCA score either. Similarly, each 25 nmol/L decrease in serum 25(OH) D level was not associated with the change in the MoCA score (beta 0.33, 95% CI −0.17 – 0.85).

**Table 4:** Relationship between seasonal-standardized serum 25(OH)D levels and MoCA score in HUNT4 70+ (n=662)

**Seasonal-standardized serum 25(OH)D (nmol/L)**	**Participants (n)**	**MoCA score mean (range)**	**Crude β (95% CI)**	**Adjusted β (95% CI) Model 1†**	**Adjusted β (95% CI) Model 2¥**
Category 1					
Average <50	298	22.7 (7 – 30)	−0.23 (−0.89 – 0.43)	-0.06 (-0.67 - 0.56)	−0.02 (−0.64 – 0.60)
Average ≥50	364	22.9 (5 – 30)	0 (Reference)	0 (Reference)	0 (Reference)
Category 2					
Long-term insufficiency <50	196	22.6 (7 – 30)	−0.28 (−1.09 – 0.53)	0.04 (-0.72 - 0.79)	0.11 (−0.66 – 0.88)
Long-term sufficiency ≥50	245	22.9 (6 – 30)	0 (Reference)	0 (Reference)	0 (Reference)
Varying levels	221	22.9 (5 – 30)	−0.06 (−0.84 – 0.73)	-0.09 (-0.81 - 0.63)	−0.10 (−0.84 – 0.63)
Continuous‡	662	22.8 (5 – 30)	0.10 (−0.44 – 0.64)	0.33 (-0.17 - 0.85)	0.38 (−0.15 – 0.90)

25(OH)D, 25-hydroxyvitamin D; 95% CI, 95% confidence interval; β, coefficient; MoCA, Montreal Cognitive Assessment; †Model 1 was adjusted for age, sex, body mass index, occupation, marital status, smoking status in packyears, alcohol consumption and physical activity; ¥ Model 2 was adjusted for diabetes, blood pressure, cholesterol, depression, and kidney function(creatinine) in addition to Model 1; †per 25 nmol/L decrease in average serum 25(OH)D.

We explored the association between serum 25(OH)D classified into four categories (nmol/L, <30, 30–49.9, 50–74.9, ≥75) and cognitive function, and the results are presented in Supplementary table 1. The risk for NCDs tended to be higher in the 25(OH)D <30 nmol/L group, but the estimates were imprecise, with wide 95% CIs.

Finally, analysis of the imputed data for missing values in covariates at baseline showed similar results to the original results (Supplementary table 3 vs. Table 2).

## Discussion

### Main Findings

Our results suggested that vitamin D insufficiency assessed by an average of two serum 25(OH)D measurements with a 10-year interval was not associated with the risk of NCDs in a cohort of older Norwegian adults. A similar result was observed for long-term changes in serum 25(OH)D levels. There was no association between 25(OH)D and MoCA scores in a subsample of participants evaluated with MoCA.

### Comparison with Other Studies

Our results are consistent with some of the previous studies. In an 18-year follow-up Swedish study, Olsson et al. found no evidence for the association between baseline 25(OH)D and long-term risk of dementia or cognitive impairment (15). The null association was also reported in other studies with shorter follow-up duration (16, 17, 35). Our findings are consistent with evidence from Mendelian randomization studies using genetic variants for vitamin D levels as instruments. Two Mendelian randomization studies did not suggest a causal association between 25(OH)D and cognitive performance or dementia (18, 36). Likewise, a meta-analysis study of randomized control trials did not provide evidence that vitamin D supplementation enhances adult cognitive function (19). Similar to our findings, vitamin D supplementation failed to improve cognitive decline, measured by MoCA scores in a clinical trial (37). Overall, randomized control trials did not show a protective effect of vitamin D on cognition.

In contrast, several prospective studies have reported an association between lower 25(OH)D levels and the risk of all-cause dementia or cognitive decline (9, 10, 11, 12, 38). Sommer et al., in their meta-analysis of five prospective studies, showed an association between <25 nmol/L of 25(OH)D and an increased risk of dementia, but the relationship with 25(OH)D level of 25–50 nmol/L was unclear (13). A follow-up meta-analysis by Jayedi et al. demonstrated a continuously decreased risk of dementia with increasing 25(OH)D levels from 12.5 up to 75 nmol/L, but a protective effect could not be clarified in 25(OH) D levels ≥75 nmol/L (14).

Some factors could explain the inconsistencies in results between our study and previous prospective studies (9, 10, 11, 12, 38). Firstly, our sample size was relatively small. We observed that the risk for NCDs tended to be higher in the 25(OH)D <30 nmol/L group than in the reference group (50–74.9 nmol/L), but the difference was not statistically significant partly due to insufficient statistical power (Supplementary table 1). Secondly, we measured serum 25(OH)D twice, which better reflected 25(OH)D levels over time in the body.

### Utilizing Repeated Measures of 25(OH)D in Cognitive Health Studies

The lack of repeated 25(OH)D measurements is considered a significant limitation for previous studies. The one-time measurement is likely to introduce misclassification (13, 14, 17). Although it was suggested that 25(OH)D status could be stable over both a 5- and 14-year period (39, 40), our study showed about 33% (239/717) of participants with varying 25(OH)D levels over ten years. We observed a general increase in the mean 25(OH)D level from HUNT2 to HUNT3 after considering seasonal variations in this older Norwegian population. Even if several factors affect the circulating 25(OH) D concentrations, this increase could be attributed to more supplementation in the older group (41).

### Strength and Weakness

This prospective study is the first to evaluate the relationship between 25(OH)D and the risk of NCDs in late life using repeated measures of 25(OH)D. The two-time 25(OH)D measurements ensured an optimal reflection of vitamin D status over time to minimize exposure misclassification. NCDs in this study were diagnosed by clinical experts according to the DSM- 5 criteria following rigorous clinical assessment (3). Furthermore, we adjusted for a large panel of confounders.

There are also some limitations to the study. Firstly, we cannot entirely exclude selection bias. Among the 10% random sample of HUNT2 participants, those who did not participate in HUNT3 were older, less educated, and more likely to have chronic diseases than those who participated (25). However, our analysis cohort (n=717, Figure 1) showed similar characteristics when compared with the study cohort of participants invited for HUNT4 70+ with serum 25(OH)D in both HUNT2 and HUNT3 (n=908, Figure 1), indicating no significant selection bias (Supplementary table 2). Secondly, there could be misclassification due to measurement error in the 25(OH)D measurements, which is likely to be nondifferential in a prospective cohort study. It would be ideal to have multiple measurements of 25(OH)D levels between the 10-year period instead of the two-time measurements to minimize misclassification. Thirdly, although our study had a relatively long follow-up period of 10 years and we initially excluded three dementia cases diagnosed from 1995 to 2011, we still cannot completely exclude the possibility of reverse causation as the development of cognitive impairment could start 20–30 years before diagnosis (42). Those who had early signs of NCDs might have been advised to take vitamin D supplements, systematically increasing 25(OH)D levels in this group and potentially resulting in the null associations in the current study. Fourthly, there is a possibility of unmeasured confounding or residual confounding by missing information on confounders, though multiple imputation analysis did not suggest major bias by missing data. Lastly, our analysis cohort lacked statistical power to investigate the risk of NCDs in the deficient, 25(OH)D <30 nmol/L. Thus, we could not examine the potential nonlinear association between 25(OH)D and NCDs.

## Conclusion

Vitamin D insufficiency, defined by two times measurements of serum 25(OH)D with a 10-year interval, showed no significant impact on the risk of NCDs in a cohort of older Norwegian adults. Future studies should focus on using multiple measurements that reflect the trajectory of 25(OH) D over time with a longer follow-up duration. Studies with a larger sample size are also warranted to investigate a potential nonlinear association between 25(OH)D and cognitive function and the different types of dementia.

## Copyright comment

corrected publication in 2023

## Supplementary Material

Supplementary material, approximately 95.2 KB.

*Author Contribution:* EOA, XMM, and YQS performed literature search and contributed to the study design. XMM, HKS, GS, and YQS were responsible for data collection. EOA and YQS conducted statistical analyses, interpreted results, and wrote the initial draft of the manuscript. EOA, XMM, RSE, HKS, MK, BMB, GS, YC, and YQS participated in the data interpretation, and the manuscript writing with important intellectual content and approved the final version of the manuscript.

*Funding:* The project was supported by the Dam Foundation (Project ID: FO347301) through the Norwegian Health Association (Project ID: 19474). YQS was supported by a Researcher grant from The Liaison Committee for education, research, and innovation in Central Norway (project ID 2018/42794). The funders had no role in study design, data collection, analysis, decision to publish, or manuscript preparation. The corresponding authors had access to all the data in the study and had final responsibility for the decision to submit for publication. NTNU Norwegian University of Science and Technology (incl St. Olavs Hospital - Trondheim University Hospital). Open access funding provided by NTNU Norwegian University of Science and Technology (incl St. Olavs Hospital - Trondheim University Hospital).

*Data availability statement:* Data from the HUNT Study used in research projects will, when reasonably requested by others, be made available on request to the HUNT Data Access Committee. The HUNT data access information describes the policy regarding data availability (https://www.ntnu.edu/hunt/data).

*Ethics declarations:* The study was approved by the Norwegian Regional Committees for Medical and Health Research Ethics (no. 31812). All HUNT participants have signed informed consent for participation and the use of data in research.
