# Supplementary material for: Vitamin D Status Over Time and Cognitive Function in Norwegian Older Adults: A Prospective Cohort of the HUNT Study
Source: J Nutr Health Aging. 2022 Dec 7;27(1):30–7. doi: 10.1007/s12603-022-1867-8 (PMC12880073; doi:10.1007/s12603-022-1867-8)

**SUPPLEMENTARY MATERIAL**

**Contents**

**Supplementary table 1:** Relationship between average seasonal-standardized serum 25(OH)D level of HUNT2 and HUNT3 and risk of NCDs in HUNT4 70+ (n=717)

**Supplementary table 2**: Baseline characteristics in the HUNT2 survey for the study cohort invited for HUNT4 70+ and the analysis cohort

**Supplementary table 3:** Relationship between average seasonal-standardized serum 25(OH)D level of HUNT2 and HUNT3 and risk of NCDs in HUNT4 70+ (n=717) Analyses after performing multiple imputation of missing data in covariates

**Supplementary figure 1:** Box plot reflecting seasonal-standardized serum 25(OH)D variability in HUNT2 & HUNT3

**Supplementary figure 2:** Scatter plot of serum 25(OH)D and MoCA score (n=662)

**Supplementary table 1**

Relationship between average seasonal-standardized serum 25(OH)D level of HUNT2 and HUNT3 and risk of NCDs in HUNT4 70+ (n=717)

| Average seasonal-standardized serum 25(OH)D (nmol/L) | Participants (n) | Cases (n) | Risk (%) | Crude OR (95% CI) | Adjusted OR (95% CI) Model 1^†^ | Adjusted OR (95% CI) Model 2^¥^ |
| --- | --- | --- | --- | --- | --- | --- |
| NCDs* | | | | | | |
| <30 | 22 | 13 | 59.1 | 1.62 (0.67 – 3.88) | 1.31 (0.51 – 3.38) | 1.23 (0.46 – 3.24) |
| 30–49.9 | 302 | 149 | 49.3 | 1.09 (0.80 – 1.49) | 1.06 (0.75 – 1.49) | 1.01 (0.71 – 1.43) |
| 50–74.9 | 339 | 160 | 47.2 | 1.00 (Reference) | 1.00 (Reference) | 1.00 (Reference) |
| ≥75 | 54 | 25 | 46.3 | 0.96 (0.54 – 1.72) | 1.14 (0.61 – 2.13) | 1.10 (0.58 – 2.06) |
| Mild Cognitive impairment (MCI) | | | | | | |
| <30 | 22 | 9 | 40.9 | 1.56 (0.60 – 4.04) | 1.36 (0.49 – 3.75) | 1.27 (0.45 – 3.56) |
| 30–49.9 | 302 | 100 | 33.1 | 1.02 (0.72 – 1.44) | 0.98 (0.68 – 1.42) | 0.95 (0.65 – 1.38) |
| 50–74.9 | 339 | 115 | 33.9 | 1.00 (Reference) | 1.00 (Reference) | 1.00 (Reference) |
| ≥75 | 54 | 15 | 27.8 | 0.81 (0.41 – 1.57) | 0.94 (0.46 – 1.89) | 0.89 (0.44 – 1.81) |
| Dementia | | | | | | |
| <30 | 22 | 4 | 18.2 | 1.77 (0.52 – 6.00) | 1.13 (0.28 – 4.64) | 1.05 (0.24 – 4.56) |
| 30–49.9 | 302 | 49 | 16.2 | 1.27 (0.81 – 2.02) | 1.26 (0.74 – 2.14) | 1.19 (0.69 – 2.07) |
| 50–74.9 | 339 | 45 | 13.3 | 1.00 (Reference) | 1.00 (Reference) | 1.00 (Reference) |
| ≥75 | 54 | 10 | 18.5 | 1.37 (0.62 – 3.02) | 1.87 (0.75 – 4.66) | 1.96 (0.78 – 4.96) |

25(OH)D, 25-hydroxyvitamin D; 95% CI, 95% confidence interval; NCDs, neurocognitive disorders; OR odds ratio.

*NCDs consisted of mild cognitive impairment (MCI) and dementia.

^†^Model 1 was adjusted for age, sex, body mass index, occupation, marital status, smoking status in packyears, alcohol consumption and physical activity.

^¥^Model 2 was adjusted for diabetes, blood pressure, cholesterol, depression, and kidney function (creatinine) in addition to Model 1.

**Supplementary table 2**

Baseline characteristics in the HUNT2 survey for the study cohort invited for HUNT4 70+ and the analysis cohort

|  | Study cohort invited for HUNT4 70+ | Analysis cohort |
| --- | --- | --- |
|  | (n=908) | (n=717) |
|  |  |  |
| Age (years) | 56.0 ± 6.3 | 55.9 ± 6.2 |
|  |  |  |
| Sex |  |  |
| Male | 409 (45.0) | 322 (44.9) |
| Female | 499 (55.0) | 395 (55.1) |
|  |  |  |
| Body mass index (kg/m^2^) |  |  |
| Underweight or Normal (<25.0) | 279 (30.7) | 216 (30.1) |
| Overweight (25.0–29.9) | 468 (51.5) | 374 (52.2) |
| Obesity (≥30.0) | 159 (17.5) | 125 (17.4) |
| Unknown | 2 (0.2 | 2 (0.3) |
|  |  |  |
| Occupation^†^ |  |  |
| EGP Class I | 85 (9.4) | 71 (9.9) |
| EGP Class II | 121 (13.3) | 100 (14.0) |
| EGP Class III | 162 (17.8) | 131(18.3) |
| EGP Class IV | 153 (16.9) | 125 (17.4) |
| EGP Class V + VI | 62 (6.8) | 49 (6.8) |
| EGP Class VII | 104 (11.5) | 81 (11.3) |
| Unknown | 221 (24.3) | 160 (22.3) |
|  |  |  |
| Marital status |  |  |
| Unmarried | 43 (4.7) | 29 (4.0) |
| Married | 748 (82.4) | 596 (83.1) |
| Others* | 117 (12.9) | 92 (12.8) |
|  |  |  |
| Smoking status in packyears (pyrs) |  |  |
| Never smokers | 413 (45.5) | 335 (46.7) |
| Former smokers 0–10 pyrs | 137 (15.1) | 111 (15.5) |
| Former 10.1–20 pyrs | 64 (7.0) | 51 (7.1) |
| Former >20 pyrs | 46 (5.1) | 37 (5.2) |
| Current smokers 0–10 pyrs | 41 (4.5) | 29 (4.0) |
| Current 10.1–20 pyrs | 66 (7.3) | 53 (7.4) |
| Current >20 pyrs | 72 (7.9) | 52 (7.3) |
| Unknown | 69 (7.6) | 49 (6.8) |
|  |  |  |
| Alcohol consumption |  |  |
| Never | 323 (35.6) | 246 (34.3) |
| 1–4 times per month | 392 (43.2) | 314 (43.8) |
| ≥ 5 times per month | 119 (13.1) | 98 (13.7) |
| Unknown | 74 (8.1) | 59 (8.2) |
|  |  |  |
| Physical activity |  |  |
| Inactive | 176 (19.4) | 134 (18.7) |
| Low | 146 (16.1) | 115 (16.0) |
| Moderate | 182 (20.0) | 151 (21.1) |
| High | 82 (9.0) | 69 (9.6) |
| Unknown | 322 (35.5) | 248 (34.6) |
|  |  |  |
| Diabetes |  |  |
| No | 882 (97.1) | 698 (97.4) |
| Yes | 23 (2.5) | 17 (2.4) |
| Unknown | 3 (0.3) | 2 (0.3) |
|  |  |  |
| Hypertension |  |  |
| No | 470 (51.8) | 380 (53.0) |
| Yes | 438 (48.2) | 337 (47.0) |
|  |  |  |
| Serum cholesterol (mmol/L) |  |  |
| Desirable (<5.2) | 156 (17.2) | 124 (17.3) |
| Borderline (5.2 – 6.2) | 326 (35.9) | 256 (35.7) |
| High (>6.2) | 425 (46.8) | 336 (46.9) |
| Unknown | 1 (0.1) | 1 (0.1) |
|  |  |  |
| Depression (HADS) |  |  |
| Non cases (≤7) | 779 (85.8) | 617 (86.1) |
| Cases (≥8) | 79 (8.7) | 61 (8.5) |
| Unknown | 50 (5.5) | 39 (5.4) |
|  |  |  |
| Serum creatinine [µmol/L] | 69.9 ± 13.0 | 69.6 ± 12.6 |

Data are given as the number of participants (column percentage) or mean ± 1SD.

25(OH)D, 25-hydroxyvitamin D; EGP, Erikson Goldthorpe Portocarero social class scheme; HUNT, Trøndelag Health Study; HADS, The Hospital Anxiety and Depression Scale.

^†^Occupation: EGP Class I (administrative managers, politicians or academic professions), EGP Class II (occupations with shorter college and university degrees), EGP Class III (office and customer service occupations, sales, service and care professions), EGP Class IV (occupations in agriculture, forestry and fishing), EGP Class V + VI (craftsmen, process and machine operators or transport), EGP Class VII (occupations without education requirements).

*Others (widow/widower, divorced, separated and unknown).

**Supplementary table 3**

Relationship between average seasonal-standardized serum 25(OH)D level of HUNT2 and HUNT3 and risk of NCDs in HUNT4 70+ (n=717)

Analyses after performing multiple imputation of missing data in covariates

| Average seasonal-standardized serum 25(OH)D (nmol/L) | Participants (n) | Cases (n) | Risk (%) | Crude OR (95% CI) | Adjusted OR (95% CI) Model 1^†^ | Adjusted OR (95% CI) Model 2^¥^ |
| --- | --- | --- | --- | --- | --- | --- |
| NCDs^*^ | | | | | | |
| Categorical |  |  |  |  |  |  |
| <50 | 324 | 162 | 50.0 | 1.12 (0.84 – 1.51) | 1.05 (0.76 – 1.46) | 1.04 (0.75 – 1.46) |
| ≥50 | 393 | 185 | 47.1 | 1.00 (Reference) | 1.00 (Reference) | 1.00 (Reference) |
| Continuous^ⱡ^ | 717 | 347 | 48.4 | 1.09 (0.86 – 1.38) | 0.96 (0.73 – 1.26) | 0.95 (0.72 – 1.26) |
|  |  |  |  |  |  |  |
| Mild cognitive impairment (MCI) | | | | | | |
| Categorical |  |  |  |  |  |  |
| <50 | 324 | 109 | 33.6 | 1.08 (0.78 – 1.49) | 0.99 (0.69 – 1.41) | 0.98 (0.68 – 1.42) |
| ≥50 | 393 | 130 | 33.1 | 1.00 (Reference) | 1.00 (Reference) | 1.00 (Reference) |
| Continuous^ⱡ^ | 717 | 239 | 33.3 | 1.17 (0.89 – 1.53) | 1.04 (0.76 – 1.41) | 1.03 (0.75 – 1.42) |
|  |  |  |  |  |  |  |
| Dementia | | | | | | |
| Categorical |  |  |  |  |  |  |
| <50 | 324 | 53 | 16.4 | 1.24 (0.81 – 1.90) | 1.21 (0.73 – 2.01) | 1.21 (0.73 – 2.02) |
| ≥50 | 393 | 55 | 14.0 | 1.00 (Reference) | 1.00 (Reference) | 1.00 (Reference) |
| Continuous^ⱡ^ | 717 | 108 | 15.1 | 0.94 (0.67 – 1.32) | 0.81 (0.53 – 1.23) | 0.79 (0.52 – 1.21) |

25(OH)D, 25-hydroxyvitamin D; 95% CI, 95% confidence interval; NCDs, neurocognitive disorders; OR, odds ratio.

^*^NCDs consisted of mild cognitive impairment (MCI) and dementia.

^†^Model 1 was adjusted for age, sex, body mass index, occupation, marital status, smoking status in packyears, alcohol consumption and physical activity.

^¥^Model 2 was adjusted for diabetes, hypertension, serum cholesterol, depression, and kidney function (serum creatinine) in addition to Model 1

**^ⱡ^**per 25 nmol/L decrease in serum 25(OH)D.**Supplementary figure 1**

Box plot reflecting seasonal-standardized serum 25(OH)D variability in HUNT2 & HUNT3


25(OH)D, 25-hydroxyvitamin D

**Supplementary figure 2**

Scatter plot of serum 25(OH)D and MoCA score (n=662)

MoCA, Montreal Cognitive Assessment; the fitted line is derived from linear regression
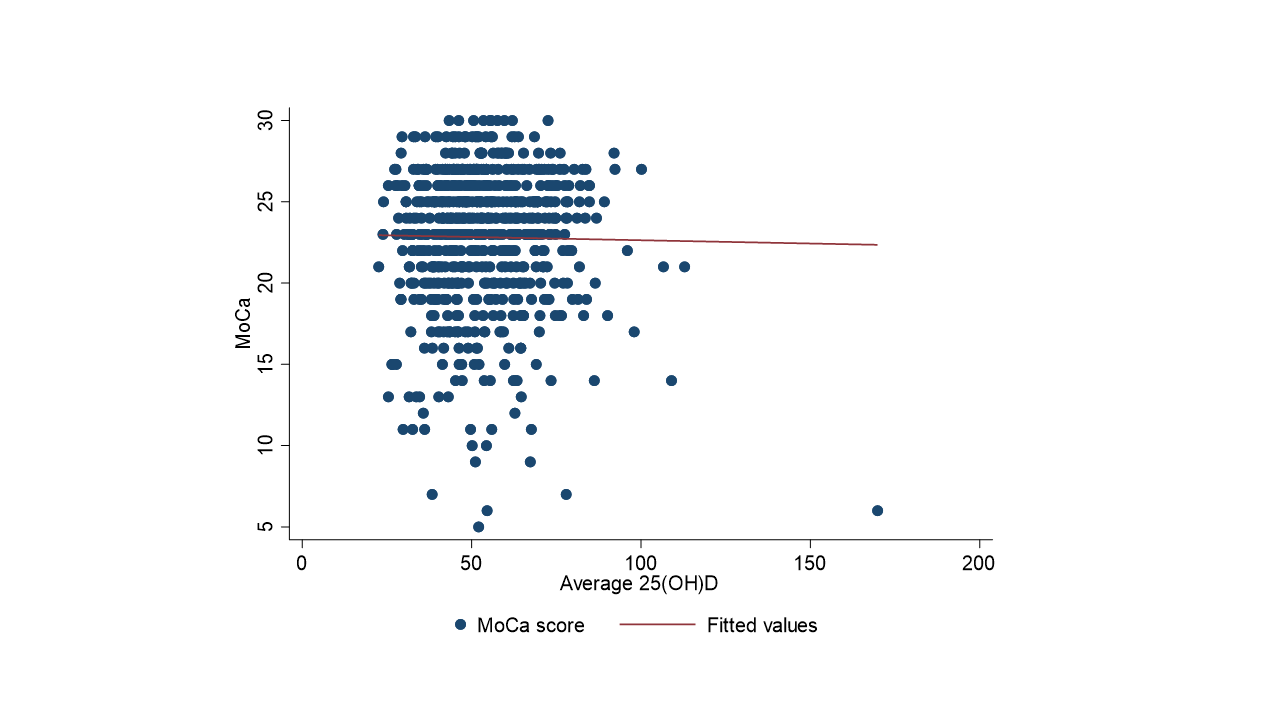

Supplement: Supplementary file 1 — Supplementary material, approximately 95.2 KB. [file mmc1.docx]
